# Parvalbumin interneurons contribute to spontaneous hemodynamic fluctuations

**DOI:** 10.1177/0271678X261462775

**Published:** 2026-07-15

**Authors:** Adiya Rakymzhan, Mitsuhiro Fukuda, Alberto L. Vazquez

**Affiliations:** 1 Department of Bioengineering, University of Pittsburgh, Pittsburgh, PA, USA; 2 Center for the Neural Basis of Cognition, University of Pittsburgh and Carnegie Mellon University, Pittsburgh, PA, USA; 3 Department of Radiology, University of Pittsburgh, Pittsburgh, PA, USA; 4 Center for Neuroscience, University of Pittsburgh, Pittsburgh, PA, USA

**Keywords:** Resting state, low-frequency hemodynamic fluctuations, gamma oscillations, inhibitory neurons, neurovascular coupling

## Abstract

Resting-state hemodynamic fluctuations take place over broad temporal scales and are reported to be closely linked to gamma-band neural activity, yet whether specific cell types drive these fluctuations remains unclear. Given the established contribution of parvalbumin (PV) neurons in generating gamma oscillations, they are prime candidates. Using chemogenetic tools in awake PV-Cre mice, we modulated PV interneuron activity and measured effects on neural network activity, EEG, and hemodynamics. Two-photon calcium imaging confirmed their modulation of excitatory neurons as well as local vascular changes. PV chemogenetic suppression reduced EEG gamma power, increased low-frequency EEG activity, and elevated basal CBF. Two-photon imaging under control conditions showed increased basal arterial diameter and significantly greater vascular fluctuation in deeper cortical layers enriched with PV cells, but not in superficial layers. Importantly, PV suppression significantly weakened the correlation between the EEG gamma power and CBF. These findings provide evidence that PV interneuron contribute to spontaneous neurovascular dynamics and to the link between gamma oscillations and resting-state hemodynamic signals, pointing to a significant role in neurovascular changes during non-task-engaged brain states.

## Introduction

Spontaneous fluctuations in hemodynamic signals, particularly at frequencies around 0.1 Hz, are commonly observed during so-called resting state imaging experiments.^1–4^ This term typically refers to a condition where a subject lies in the functional magnetic resonance imaging (fMRI) scanner without performing an explicit task or being exposed to a specific stimulus. Studies using fMRI and intrinsic optical imaging have revealed that these low-frequency fluctuations are present in animals and humans and they are strongly synchronized within similar networks and across distant, functionally connected regions.^1–8^ This phenomenon, known as “functional connectivity,” provides valuable insights into the brain’s functional organization and underscore the utility of hemodynamic-based imaging techniques for studying brain function.^2,6,9,10^

These hemodynamic low-frequency fluctuations are associated with neural activity in areas with known anatomical connections.^11^ To capture activity over slower hemodynamic timescales, which typically lag neural activity by a few seconds, researchers have used changes in band-limited power (BLP) derived from LFP measurements.^12^ These BLP changes not only reflect large-amplitude fluctuations over extended time scales but also show synchronization across distant cortical regions, linking local neuronal activity to broader network dynamics.^12^ In monkeys, concurrent LFP-fMRI recordings have revealed that spontaneous BOLD fluctuations strongly correlate with BLP in the gamma (30–100 Hz) frequency range.^13,14^ Similarly, in mice, low-frequency arterial oscillations have been found to phase-lock with slow variations in gamma BLP.^4,8,15^ Together, these findings highlight a robust relationship between neural gamma activity and spontaneous hemodynamic fluctuations, though the mechanisms underlying this link remain unclear.

Gamma activity in the cortex is thought to have a strong contribution from parvalbumin (PV) interneuron activity. These interneurons provide strong inhibitory input to local populations of asynchronously firing principal cells, effectively suppressing their activity and generating synchronized gamma rhythms.^16^ Optogenetic studies have demonstrated the critical role of PV interneurons in gamma oscillation generation, where selective activation of PV cells induced gamma rhythms and their inhibition attenuated them significantly.^17,18^ Given the strong correlation between gamma BLP and spontaneous hemodynamic fluctuations, it is plausible that PV interneurons not only drive gamma activity but also play a key role in regulating resting-state cerebral blood flow (CBF) dynamics.

Neurons regulate CBF by manipulating the tone of nearby blood vessels, a process known as neurovascular coupling (NVC).^19^ Different neuronal populations exert distinct effects on vascular dynamics.^20–23^ Advances in genetic tools and the emergence of optogenetics have enabled researchers to dissect the contributions of specific neuronal subpopulations on CBF regulation. In vivo optogenetic studies in anesthetized mice have shown that activating GABAergic inhibitory neurons induces a large increase in CBF.^24,25^ Among inhibitory neurons, our previous work in awake mice showed that activating PV interneurons produces slow, gradual vasodilations, particularly in deeper cortical layers where these neurons are predominantly concentrated.^23,26^ Notably, these slow vasodilations closely resemble low-frequency, high-amplitude hemodynamic fluctuations observed in rodents and humans under resting state conditions. While studies on evoked activity of PV neurons have provided valuable insights into their influence on CBF, their role in regulating spontaneous CBF fluctuations during awake resting-state activity remains unclear.^26–30^ Correlational analyses suggest that synchronized PV cell activity drives depth-specific vascular changes, but more causal evidence is lacking.^26^

In this study, we sought to evaluate the role of PV interneurons on CBF regulation under resting state conditions by selectively modulating their activity using chemogenetics. To achieve this, we used transgenic PV-cre mice to express a cre-dependent Designer Receptor Exclusively Activated by Designer Drugs (DREADD) to selectively modulate PV interneurons in the primary somatosensory cortex (S1). We first assessed the contribution of PV interneurons to network dynamics by imaging calcium activity in PV-positive (PV+) and PV-negative (PV−) cells using two-photon laser scanning microscopy (2PLSM). We also measured neuronal activity using electroencephalogram (EEG) at baseline and after DREADD activation. We then evaluated how PV interneuron modulation affects spontaneous hemodynamic fluctuations by measuring CBF changes with Laser Doppler flowmetry (LDF) and vessel tone with 2PLSM. Finally, we investigated the impact of PV suppression on the coupling between neural activity and hemodynamic oscillations under resting state conditions. Our findings indicate that PV interneurons significantly modulate ongoing network activity, spontaneous hemodynamic fluctuations and NVC. These results suggest that PV cell signaling significantly contributes to the underlying strong correlation between gamma oscillations and CBF during resting-state conditions.

## Materials and methods

### Experimental model

We used a total of 12 adult transgenic mice (male *n* = 7, female *n* = 5) expressing cre-recombinase in PV neurons (strain B6.129P2-Pvalb(tm1-cre)Arbr/J), purchased from The Jackson Laboratory (Bar Harbor, ME USA). All procedures performed in accordance with a protocol approved by the University of Pittsburgh Institutional Animal Care and Use Committee (IACUC) and followed standards for humane animal care and use as set by the Animal Welfare Act (AWA), the National Institute of Health Guide for the Care and Use of Laboratory Animals and ARRIVE (Animal Research: Reporting in Vivo Experiments).

### Animal surgery for awake head-fixed experiments

**Figure 1.**
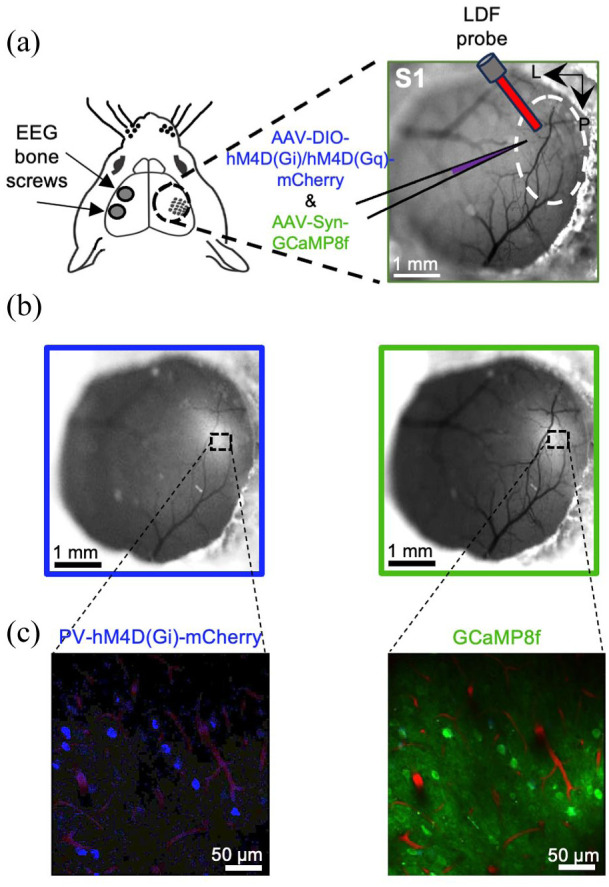
Cranial window and viral expression in S1 of a PV-cre mouse: (a) cranial window implanted in S1 of a PV-cre mouse, where we injected inhibitory DREADD (AAV-DIO-hM4D(Gi)-mCherry) and calcium reporter (AAV-Syn-GCaMP8f) viruses. Two metal EEG bone screws were implanted in the contralateral hemisphere for EEG recordings. LDF probe was placed on the cover glass, avoiding large vessels. (b) Surface fluorescence microscope images below showing the regions expressing hM4D(Gi)-mCherry (blue box) and GCaMP8f (green box). (c) 2PLSM microscopy images of S1 intra-cortical sections (~250 μm depth) showing the regions expressing hM4D(Gi)-mCherry (blue cells) and GCaMP8f (green cells). Vessel contrast enhanced by Sulforhodamine 101 injection.

When mice were 8–10 weeks of age, we injected cre-dependent AAV virus encoding inhibitory DREADD-mCherry (AAV-hSyn-DIO-hM4D(Gi)-mCherry; University of North Carolina Vector Core, Chapel Hill, NC USA) in S1 (Figure 1(a)–(c)). To drive the expression of a calcium (Ca^2+^) reporter in neurons, the injection included a secondary AAV virus targeting the Synapsin promoter (AAV-Syn-GCaMP7f or AAV-Syn-GCaMP8f; Addgene, Watertown, MA USA) (Figure 1(a)–(c)). For the control group, we used male adult PV-cre mice injected with AAV-hSyn-DIO-mCherry and AAV-Syn-GCaMP7f or AAV-Syn-GCaMP8f.

Each animal underwent a surgical procedure to implant a cranial window for optical access (Figure 1(a)). Briefly, the exposed brain was sealed with a glass coverslip (4 mm in diameter), and a custom head-plate was fixed to the skull. Two metal bone screws (stainless steel bone screws, 0.86 mm × 4 mm, Fine Science Tools, Foster City, CA) were implanted in the opposite hemisphere for EEG recordings (Figure 1(a)). Mice were given at least 4 weeks to recover post-surgery and before imaging. During the recovery period, mice were acclimated to head fixation on a treadmill (1 h of treadmill in their cage followed by 1 h of treadmill under the microscope for 5 days) during the four weeks post-surgery. Mice were imaged at the age of 3–8 months.

### Two-photon microscopy

**Figure 2.**
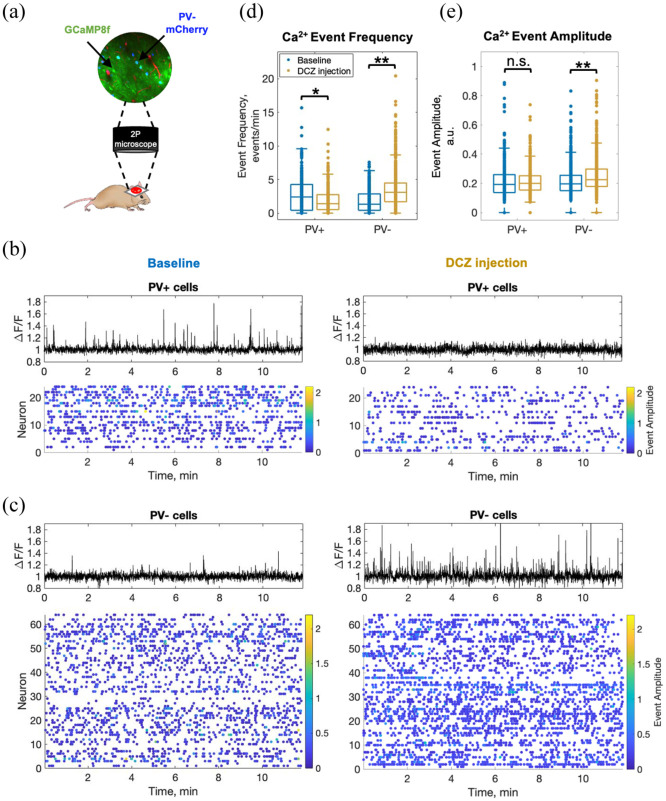
Activation of hM4Di(Gi) DREADD with DCZ effectively suppresses PV neuron activity and enhances network activity: (a) 2PLSM imaging setup, where neurons expressing GCaMP8f, including PV-mCherry cells, were visualized. (b) Time profiles of Ca^2+^ fluorescence changes (black traces) in an individual PV+ cell in a PV-hM4Di(Gi) mouse during ongoing activity at baseline and post-DCZ injection. Below are the raster plots of Ca^2+^ activity in PV+ cell population from a single animal at baseline and post-DCZ injection. Each dot represents a Ca^2+^ event and each raw represents an individual neuron. The dot color encodes an event amplitude. (c) Similar analysis as in B but for PV− cells. (d) Summary of Ca^2+^ event frequency and (e) Ca^2+^ event amplitude in PV+ and PV− cells. Cells with no detected events were assigned an amplitude of 0. Significant differences are denoted by * for *p* < 0.05 or ** for *p* < 0.01. Non-significant differences are denoted by n.s.

Each mouse underwent at least 3 awake daytime imaging sessions, with 1 week between the sessions. Before imaging, Sulforhodamine 101 (SR101; Thermo Fisher Scientific, Waltham, MA USA), dissolved in saline (5–10 mM), was injected intraperitoneally (IP; 8 μL/g body weight) to image the cerebral vasculature.^31^ Imaging was performed using a two-photon fluorescence microscope (2PLSM Plus, Bruker Nano Inc., Billerica, MA USA) equipped with an InSight X3 widely tunable ultrafast laser (Spectra-Physics, Milpitas, CA USA) and a 16X water immersion objective lens (0.80 NA; Nikon Inc., Tokyo, Japan) with a maximum field-of-view (FOV) of 1100 × 1100 μm^2^ (Figure 2(a)).

Two-photon excitation was performed at 900 nm for imaging GCaMP and SR101 in S1 cortex at depths of 0–400 μm (layers 1–4). The following imaging parameters were used to acquire time series: raster scanning rate at 2.4 μs/pixel; 332 × 332 μm^2^ FOV with 1.66 μm/pixel; 200 × 200 matrix; 5.1 Hz frame rate. We measured arteries and arterioles since traditionally they are considered the major vascular locations for blood flow regulation. The selection of the imaged region was based on three parameters, where the FOV includes: penetrating vessels, GCaMP cells, and PV-mCherry cells.

Motion artifacts in the 2P imaging data were corrected computationally by estimating per-frame *x*/*y* translational shifts via cross-correlation, applying shifts to all channels, and regressing residual motion signals from each pixel’s time course; frames with intensity deviations exceeding a set threshold were identified as motion artifacts and excluded from analysis.

### EEG and LDF recordings

**Figure 3.**
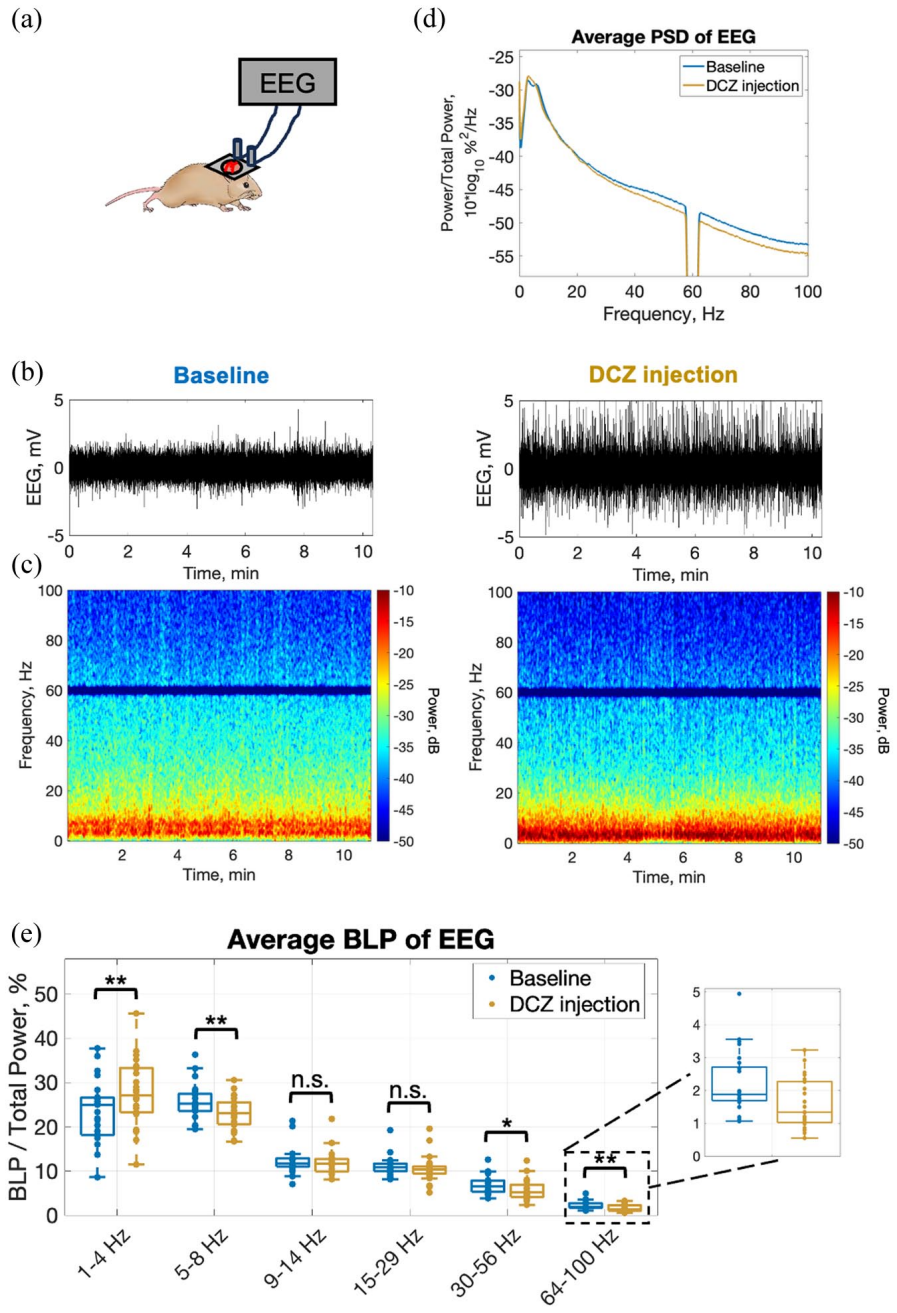
Suppression of PV neuron activity decreases high-gamma power and increases low-frequency power in EEG: (a) EEG recording setup, (b) example of EEG traces, and (c) example of EEG signal spectrogram during ongoing activity at baseline and post-DCZ injection in a PV-hM4Di(Gi) mouse. (d) Average PSD of EEG signals and (e) average BLPs of EEG in PV-hM4Di(Gi) mice during ongoing activity at baseline and post-DCZ injection. Significant differences are denoted by * for *p* < 0.05 or ** for *p* < 0.01. Non-significant differences are denoted by n.s.

**Figure 4.**
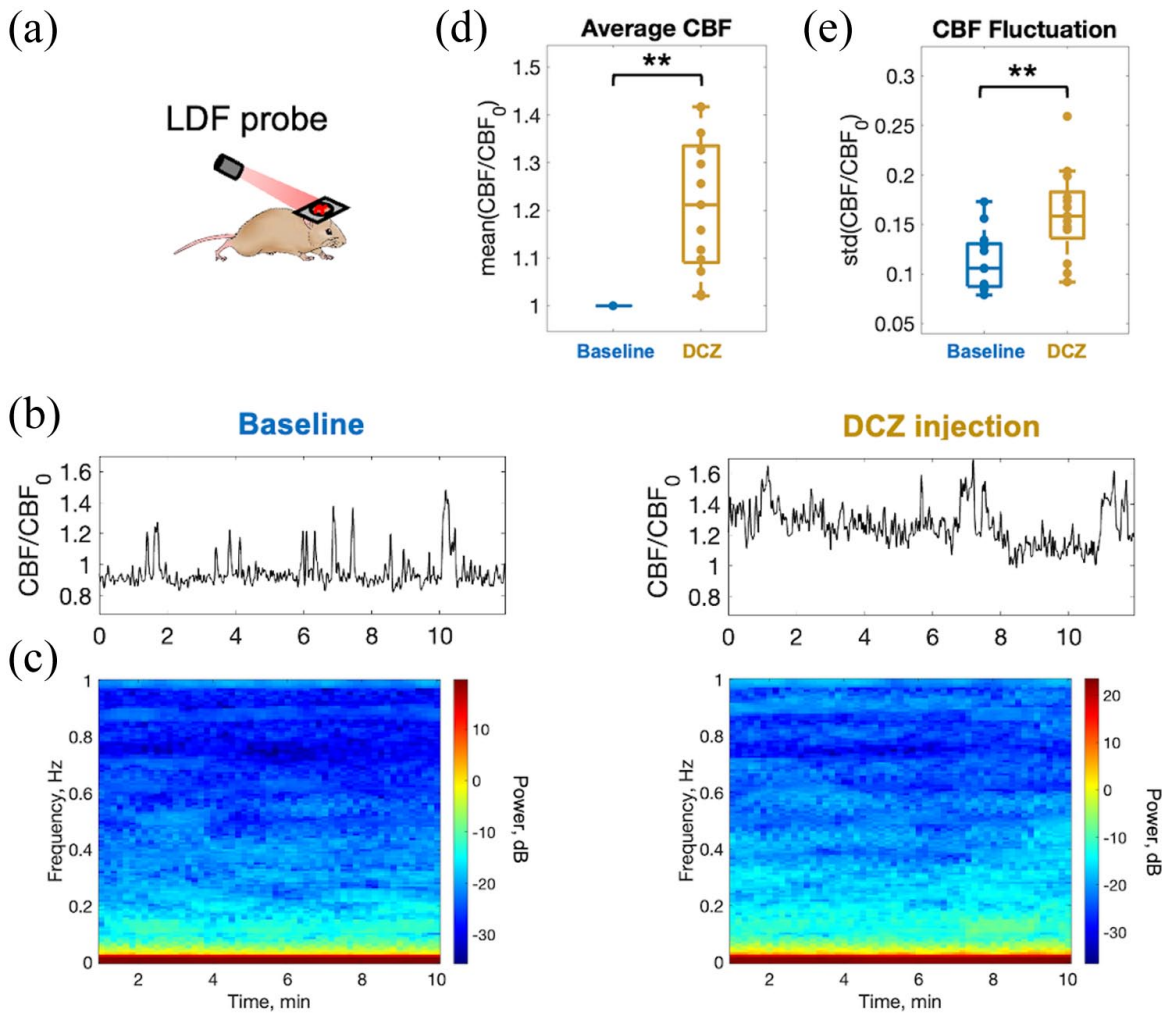
Suppression of PV neuron activity increases spontaneous CBF: (a) LDF recording setup for CBF measurements, where LDF probe was placed on top of cranial window, (b) example of spontaneous CBF time series, (c) corresponding spectrogram at baseline and post-DCZ injection in a PV-hM4Di(Gi) mouse, (d) average normalized CBF at baseline and post-DCZ injection, and (e) average CBF fluctuation amplitude at baseline and post-DCZ injection. Significant differences are denoted by ** for *p* < 0.01.

EEG was recorded with implanted bone screws (Figure 1(a), Figure 3(a)) at a frequency of 1 kHz (BIOPAC System, Inc., Goleta, CA). CBF changes were measured using LDF (Periflux 5000/411, Perimed AB, Jarfalla, Sweden) acquired at 1 kHz. The LDF probe, with a tip diameter of 450 μm and operating wavelength of 780 nm, was placed at 60º on the cover glass facing the expressing region, avoiding large surface vessels (Figure 4(a)).

Motion artifacts in EEG recordings were minimized by excluding epochs exceeding a set voltage threshold, while LDF signals were screened for sharp transient spikes exceeding a set threshold. Both EEG and LDF signals were additionally verified through visual inspection.

### Baseline recordings and DREADD activation

Following head-fixation on the treadmill, baseline activity was recorded for 30 min. To suppress PV+ interneurons by activating hM4Di(Gi) DREADD, we used DREADD agonist Deschloroclozapine (DCZ; HB85555, Hello Bio Inc., Princeton, NJ USA). 1 mg of DCZ was dissolved in 3.42 mL of 2% DMSO (prepared by diluting 100% DMSO with sterile saline) and stored at −20°C until use. For injections, aliquots were diluted with sterile saline to a working concentration of 0.1 mg/mL. Mice received IP injections of 1 μg/kg DCZ. Given the rapid onset of DCZ action, a 5-minute stabilization period was observed post-injection, followed by a 30-minute recording session under DREADD-mediated suppression.

### Data analysis

All data were analyzed using MATLAB (MathWorks, Natick, MA USA).

#### Cell region of interest (ROI)

Active cells expressing GCaMP were segmented from the average intensity 2PLSM image using a custom function in MATLAB based on temporal correlation to identify locally connected pixels. PV+ cells were identified based on mCherry expression (Figure 1(c)), and the rest of the cells were considered PV− cells. The final ROIs were qualitatively examined to match the morphological features of neurons. The Ca^2+^ fluorescence signal of each cell body was extracted by averaging all pixels within the ROI. Ca^2+^ change traces were corrected for neuropil contamination by regressing out the first two principal components of the overall neuropil Ca^2+^ trace.

#### Ca^2+^ event calculation

To infer Ca^2+^ events from the Ca^2+^ fluorescence traces, we applied OASIS (Online Active Set method to Infer Spikes) algorithm.^32^ Fluorescence signal traces, normalized by mean fluorescence signal across time (ΔF/F), were deconvolved with the “deconvolveCa” function, applying an autoregressive model of order 1 (“ar1”) with the “foopsi” method, optimized with a sparsity penalty (“lambda”) set to 0.2 to balance the detection sensitivity for event amplitudes. Baseline fluorescence optimization (“optimize_b”) was enabled to correct for potential drifts, and the minimum spike threshold (“smin”) was set to 0.05. To account for the differing kinetics of the calcium indicators used (GCaMP7f and GCaMP8f), the deconvolution algorithm was implemented using sensor-specific temporal decay constants.

#### EEG

Raw EEG signals were bandpass filtered with a Fermi filter with cutoff frequencies between 0.1 and 120 HZ with transition band of 1 Hz. Next, the signals were filtered using a notch Fermi filter with cutoff frequencies between 58 and 62 HZ and transition band of 1 Hz to remove 60 Hz power-line interference. The resulting signals were then downsampled to 200 Hz. EEG spectrograms were generated using mtspecgramc function (Chronux toolbox), using tapers [3 5], and 4-s moving window. For calculating spectral power and power spectral density (PSD), downsampled signals were segmented into 60-s epochs. The PSD of each epoch was estimated using mtspectrumc function (Chronux toolbox), using tapers [3 5]. All PSDs were averaged across epochs, normalized by the total baseline power, log-transformed, and smoothed by 50 samples. Spectral band-limited power (BLP) was calculated by summing the PSD within predefined frequency bands, dividing by the total power across all frequencies, and expressing each band’s contribution as a percentage.

#### CBF

Time series of CBF changes were obtained from the LDF data. Raw CBF signals were downsampled to 2 Hz. CBF spectrograms were generated using mtspecgramc function (Chronux toolbox), using tapers [3 5] and 120-s moving window. To compute average CBF and CBF fluctuations, each CBF time series was normalized by the baseline mean, after which the mean and standard deviation of the resulting signal were calculated, respectively.

#### Diameter measurement

Arterial diameter was measured from 2PLSM images as the full-width-at-half-maxim um of the vessel cross-sectional intensity profile. To compute average diameter and diameter fluctuation, each diameter time series was normalized by the baseline mean, after which the mean and standard deviation of the resulting signal were calculated, respectively.

#### Correlation analysis between EEG BLP and CBF

Time-resolved BLPs of EEG signals were calculated as the squared amplitude of the band-pass-filtered signal. Next, both time-resolved BLPs and normalized CBF signals were low-pass filtered using a Fermi filter with cutoff frequency of 0.1 Hz and soft transition band of 0.3 Hz. To quantify the correlation coefficient between these two signals, Pearson’s linear correlation coefficient was computed using MATLAB’s “corr’ function.

#### Hemodynamic response function (HRF) fit

The HRF was defined by a gamma function with four parameters. The gamma fit parameters were determined using “lsqnonlin” optimization routine in MATLAB to minimize the root mean squared error between the measured vessel diameter and neural input (GCaMP signal) convolved with the gamma function. The predicted vascular response was obtained for each neuron by convolving GCaMP signal with HRF.

#### Apparent synchrony calculation and its correlation with diameter

Apparent neuronal synchrony analysis was performed over overlapping 10-s time windows, considering the Ca^2+^ reporter kinetics. For each window, we computed a Pearson correlation matrix using “corrcoef” function in MATLAB for the spiking activity deconvolved with OASIS from Ca^2+^ fluorescence signals of all detected cells. To quantify synchrony, we extracted the mean of the upper triangular matrix values for each window. Only active cells (OASIS spiking probability >1%) were included in this analysis. Arterial diameter changes were low-pass filtered using a Fermi filter with cutoff frequency of 0.1 Hz and transition band of 0.3 Hz. For analyzing cross-correlation between apparent network synchrony and arterial diameter changes, cross-correlation coefficient was computed using MATLAB’s “xcorr” function.

#### Statistical analysis

Data normality was assessed using the Shapiro–Wilk test. Differences in Ca^2+^ activity, EEG spectral BLP, CBF, arterial diameter, and the Pearson correlation between EEG time-resolved BLP and CBF were evaluated between baseline and DCZ injection using a mixed-design ANOVA corrected for multiple comparison using Bonferroni correction, with significance of *p* < 0.05. Differences in the Pearson correlation of apparent neuronal synchrony and in the cross-correlation between apparent synchrony and arterial diameter were assessed between baseline and DCZ injection using a *t*-test, with the same significance thresholds. Data are reported as mean ± standard deviation throughout the manuscript and figures. No data points or animals were excluded from the analyses.

## Results

### DREADD activation modulates PV interneuron activity and alters network dynamics

We first confirmed that DREADD activation with DCZ injection effectively manipulated PV interneuron activity. Using 2PLSM, we recorded ongoing neuronal population activity in awake, head-fixed mice expressing the Ca^2+^ sensor GCaMP (Figure 2(a)). Baseline PV+ activity and population-level Ca^2+^ dynamics (deconvolved via OASIS^27^) are illustrated in Figure 2(b) and (c).

DCZ injection in PV-hM4Di(Gi) mice effectively suppressed PV+ cell activity, as evidenced by a reduction in the number of Ca^2+^ fluorescence peaks and a lower number of Ca^2+^ events. DCZ significantly reduced PV+ event frequency (from 2.71 ± 0.13 to 1.85 ± 0.09 events/min; *p* < 0.001), while event amplitude remained stable (*p* = 0.570, Figure 2(d) and (e), *n* = 390 cells, 44 recordings, 9 animals).

Consistent with the role of PV interneurons in inhibitory gating,^16^ their suppression significantly increased PV− cell frequency (from 1.78 ± 0.06 to 3.50 ± 0.10 events/min, *p* < 0.001, Figure 2(d)) and the event amplitude (from 0.21 ± 0.003 to 0.25 ± 0.004, *p* < 0.001, *n* = 806 cells, 44 recordings, 9 animals, Figure 2(e)). Control experiments confirmed that DCZ did not alter neuronal activity in the absence of DREADD expression, as DCZ injection in naïve mice had no effect on any neuronal sub-population (Supplemental Figure 1). These findings show that DCZ injection effectively activates DREADD, allowing precise manipulation of PV interneuron activity and network activity.

### Suppression of PV interneuron activity decreases high-gamma power and increases low-frequency power in EEG

Given the evidence that PV interneurons contribute to gamma activity, we hypothesized that suppressing PV neurons would reduce gamma power in EEG.^17,18^ Following DCZ injection, we observed a notable reduction in high-frequency power as calculated by the spectral BLPs. Specifically, in the high-gamma range (64–100 Hz), the BLP significantly decreased from 2.20 ± 0.89% at baseline to 1.61 ± 0.73% post-DCZ injection (*p* < 0.001, Figure 3(e)). The low-gamma range (30–56 Hz) BLP significantly decreased from 6.82 ± 1.97% at baseline to 5.62 ± 2.18% post-DCZ injection (*p* < 0.05, 25 recordings, 9 animals, Figure 3(e)).

Interestingly, suppressing PV cells also changed low-frequency spectral BLPs (Figure 3(e)). Specifically, delta (1–4 Hz) BLP significantly increased from 23.55 ± 6.67% at baseline to 27.52 ± 7.40% post-DCZ (*p* < 0.001). Theta (5–8 Hz) BLP significantly decreased from 25.57 ± 3.92% to 23.00 ± 3.51% (*p* < 0.001). On the other hand, alpha (9–14 Hz) BLP and beta (15–29 Hz) BLP remained unchanged (12.55 ± 3.40% vs. 11.87 ± 2.72%, *p* = 0.179 and 11.04 ± 2.18% vs. 10.47 ± 2.89%, *p* = 0.239 for alpha and beta BLPs, respectively, 25 recordings, 9 animals). Together, these findings demonstrate that suppressing PV interneurons decreases high-frequency power while increasing low-frequency power in EEG, suggesting that PV suppression leads to network hyperexcitability.

### Suppression of PV interneuron activity alters spontaneous CBF

Previous studies on evoked activity have shown that PV interneurons contribute to CBF regulation by inducing slow, delayed vasodilation, resembling resting-state hemodynamic signals.^20–25^ We hypothesized that PV suppression would alter spontaneous CBF fluctuations.

LDF recordings in PV-hM4Di(Gi) mice (Figure 4(a)–(c)) revealed that suppression of PV interneurons significantly increased the average normalized CBF signal from baseline (1.00, normalized) to 1.21 ± 0.14 (*p* < 0.001, Figure 4(e)) and CBF fluctuation amplitude from 0.11 ± 0.03 to 0.16 ± 0.04 (*p* < 0.001, 13 recordings, 7 animals, Figure 4(f)). These effects were local, as contralateral CBF remained unchanged (Supplemental Figure 2A). Additionally, in a control experiment with a naïve mouse (lacking DREADD), DCZ injection did not alter CBF (Supplemental Figure 2B). Overall, these results show that suppressing PV interneurons increases basal CBF and CBF fluctuation amplitude.

### Suppression of PV interneuron activity increases basal arterial diameter and depth-dependent vascular dynamics

Since suppression of PV interneurons increased basal CBF and CBF fluctuation amplitude, we next asked whether this effect was accompanied by dilation of individual arteries. Since PV interneurons are predominantly concentrated in layers 4 and 5 in S1 cortex, we further hypothesized that PV modulation would produce the largest vascular changes closer to these layers.^21^

**Figure 5.**
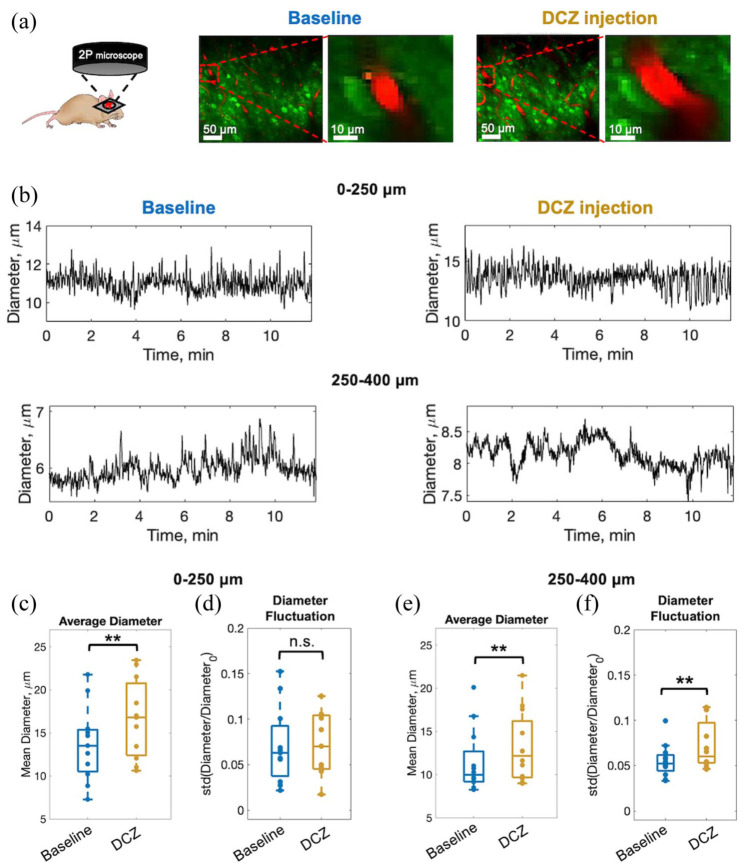
Suppression of PV neuron activity increases basal arterial diameter: (a) example 2PLSM images of an artery located ~250 μm below the cortical surface in a PV-hM4Di(Gi) mouse, (b) example time profiles of artery diameter during ongoing activity at baseline and post-DCZ injection in surface (top, 0–250 μm) and middle (bottom, 250–400 μm) layers, (c) summary of average artery diameter, (d) diameter fluctuation amplitude at baseline and post-DCZ injection in surface (e) and (f) and middle layers. Significant differences are denoted by ** for *p* < 0.01. Non-significant differences are denoted by n.s.

2PLSM imaging revealed that in surface layers (0–250 μm), suppression of PV interneurons significantly increased the average basal arterial diameter from 8.27 ± 2.51 μm to 9.95 ± 2.58 μm (*p* < 0.01, Figure 5(c)) but not the diameter fluctuation amplitude (0.07 ± 0.04 vs. 0.09 ± 0.03 (*p* = 0.307, Figure 5(d)). In middle layers (250–400 μm), we observed a similar trend in the average basal arterial diameter with the increase from 6.91 ± 2.13 μm to 7.91 ± 2.33 μm (*p* < 0.05, Figure 5(e)), as well as an increase in the diameter fluctuation amplitude from 0.06 ± 0.02 to 0.07 ± 0.03 (*p* < 0.001, 12 recordings, 6 animals, Figure 5(f)). In summary, these results show that increased basal CBF and CBF fluctuation amplitude (Figure 4) due to PV suppression is accompanied by increased basal arterial diameter across cortical depths and increased diameter fluctuation amplitude in middle layers where PV interneurons they are more numerous.

### Suppression of PV interneuron activity alters resting-state NVC

**Figure 6.**
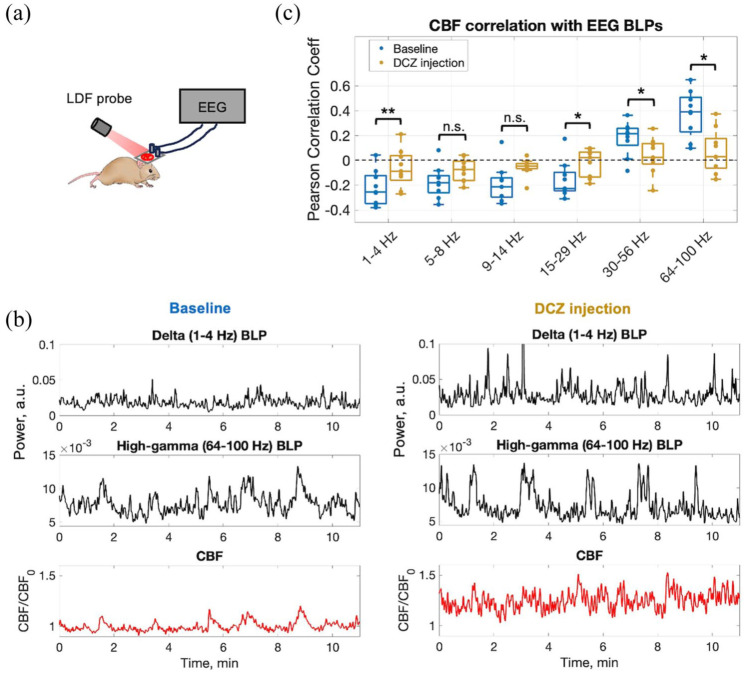
Suppression of PV neuron activity alters NVC: (a) setup for simultaneous recording of CBF and EEG using LDF and from bone screw electrodes, respectively, (b) example time profiles of delta band-limited power (1–4 Hz BLP), high-gamma band-limited power (64–100 Hz BLP), and cerebral blood flow (CBF) at baseline and post-DCZ injection in a single PV-hM4Di(Gi) mouse, (c) CBF correlation with LFP BLPs in PV-hM4Di(Gi) mice at baseline and post-DCZ injection. Significant differences are denoted by * for *p* < 0.05. Significant differences are denoted by ** for *p* < 0.01. Non-significant differences are denoted by n.s.

Previous studies in non-human primates and rodents have demonstrated a robust and consistent correlation between gamma frequency power and hemodynamic signals at rest.^4,8,13–15^ Because PV suppression alters both network activity and CBF, we next examined whether it also impacts this gamma-CBF relationship. To address this, we simultaneously recorded ongoing neural activity and CBF activity in PV-hM4Di(Gi) mice using EEG and LDF, respectively (Figure 6(a) and (b)).

Similar to spectral EEG BLP, time-resolved EEG BLP also showed a reduction in gamma and high-gamma power and increase in delta power (Supplemental Figure 3). Consistent with prior studies, our results show that CBF changes correlated most strongly with gamma (*r* = 0.178) and high-gamma (*r* = 0.379) EEG bands, whereas weaker or negative correlations were observed for delta (*r* = −0.221), theta (*r* = −0.173), alpha (*r* = −0.183), and beta (*r* = −0.158) bands (Figure 6(c)). Suppressing PV cells with DCZ significantly reduced the correlation between CBF and gamma BLP from 0.178 to 0.038 (*p* = 0.040) and high-gamma BLP from 0.379 to 0.073 (*p* < 0.01). Interestingly, PV suppression significantly reduced the magnitude of the negative correlation between CBF and BLP across delta (from −0.221 to −0.064, *p* < 0.01), theta (from −0.173 to −0.081, *p* = 0.027), alpha (from −0.183 to −0.057, *p* = 0.023), and beta (from −0.158 to −0.018, *p* < 0.001, 9 recordings, 6 animals, Figure 6(c)) frequencies. Together, these findings show that suppressing PV interneurons disrupts the strong coupling between ongoing gamma activity and spontaneous low-frequency CBF fluctuations, while attenuating the negative CBF correlation with low-frequency BLP, suggesting a frequency-specific reorganization of resting-state NVC.

### Suppression of PV interneurons enhances neuronal synchrony but not coupling to arterial dynamics

**Figure 7.**
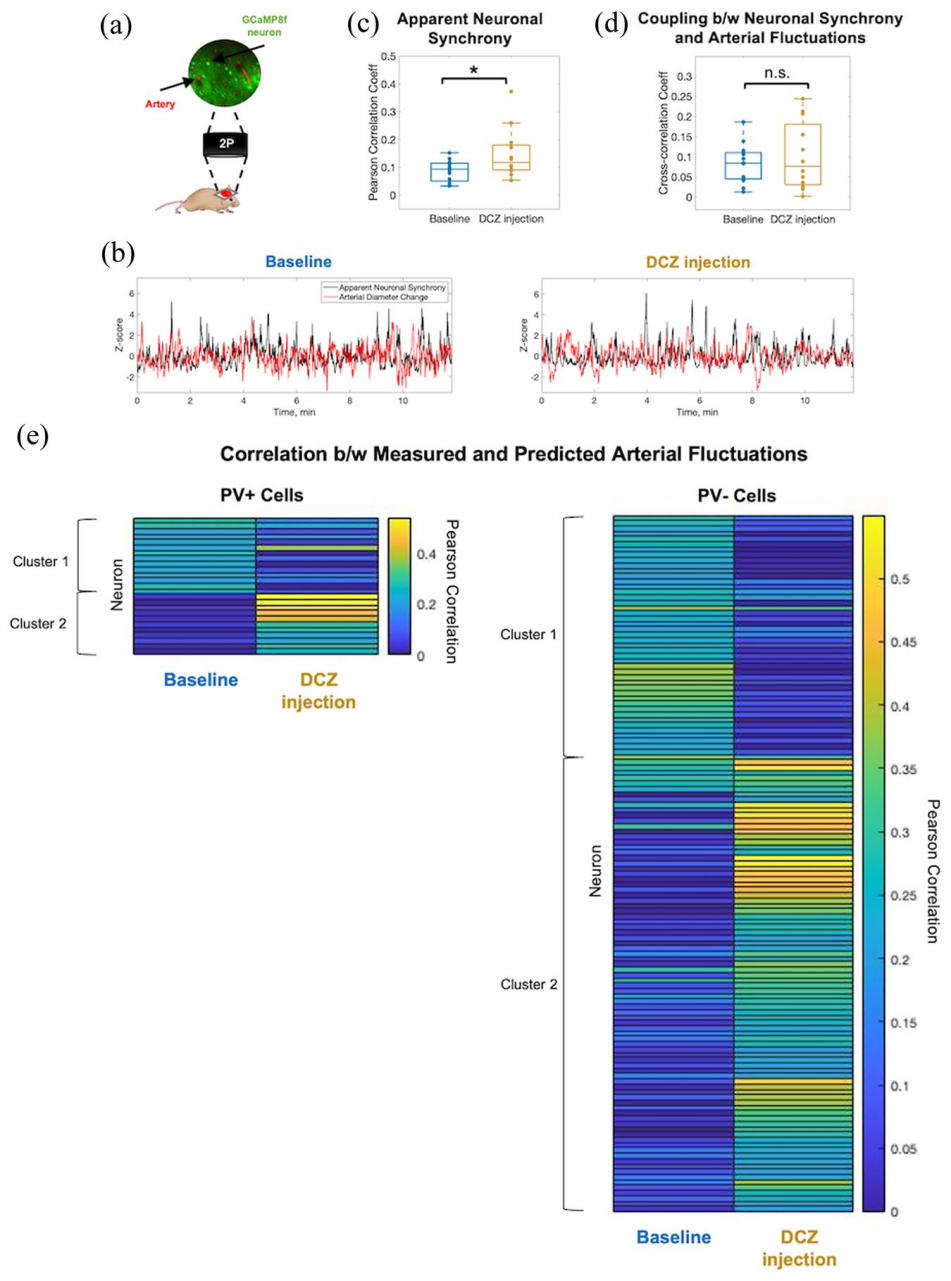
PV suppression increases neuronal synchrony without enhancing coupling to arterial dynamics: (a) schematic of the 2PLSM setup for simultaneous recording of neuronal Ca^2+^ signals (GCaMP fluorescence) and arterial diameter changes in the same field of view, (b) example time profiles of neuronal synchrony calculated as average spiking correlation between cells (black) and concurrent diameter changes of a nearby artery (red) in a PV-hM4Di(Gi) mouse at baseline and post-DCZ injection, (c) summary of apparent neuronal synchrony at baseline and post-DCZ injection, (d) summary of cross-correlation between apparent neuronal synchrony and spontaneous arterial diameter changes at baseline and post-DCZ injection, and (e) heatmaps depicting Pearson correlation coefficients between measured arterial diameter changes and predictions based on PV+ or PV− cell activity, both at baseline and post-DCZ injection. Significant differences are denoted by * for *p* < 0.05. Each row represents a single neuron, with rectangle colors indicating the magnitude of the correlation. For each neuronal subpopulation (PV+ and PV−), two clusters were identified: Cluster 1 comprises neurons with reduced correlation to arterial diameter changes after DCZ injection, while Cluster 2 includes neurons with increased correlation following DCZ injection.

Given that PV suppression produced hyper-excitability of excitatory neurons and enlarged basal arterial diameters, we examined whether the resulting hyper-excitability alters the degree of network synchrony and its temporal coordination with spontaneous vascular dynamics. To test this, we quantified the synchrony of ongoing spiking activity, estimated from Ca^2+^ fluorescence traces via OASIS deconvolution, which we called “apparent neuronal synchrony”, and related it to spontaneous arterial diameter changes (Figure 7(a)). Apparent neuronal synchrony was calculated as the average pairwise correlation of spiking activity between active neurons within a 10 s moving window under baseline conditions and post-DCZ injection in PV-hM4Di(Gi) mice (Figure 7(b), black trace). PV suppression significantly increased average inter-neuronal correlation across animals (0.087 ± 0.011 to 0.148 ± 0.025; *p* = 0.035, paired *t*-test, 12 recordings, 6 animals; Figure 7(c)), suggesting an increase in apparent neuronal synchrony.

We next examined the relationship between apparent neuronal synchrony and slow fluctuations in arterial diameter (Figure 7(b), red trace) by calculating the cross-correlation between these two signals. Average observed maximum cross-correlation coefficient at baseline conditions was 0.144 ± 0.040 (*p* = 0.010). Surprisingly, it was not significantly altered upon PV suppression (0.158 ± 0.048 post-DCZ, *p* = 0.411, paired *t*-test, 12 recordings, 6 animals, Figure 7(d)). These results suggest that despite this apparent network synchrony upon PV suppression, it does not translate into stronger coupling to arterial dynamics, and requiring normal PV neuron activity.

### PV+ and PV− cells exhibit state-dependent contributions to vascular regulation

Because PV suppression disrupted canonical network-level predictors of CBF while increasing neuronal synchrony without enhancing arterial coupling, we next asked whether neurovascular control is redistributed across individual neurons. We therefore modeled arterial dynamics from single-cell activity to determine how cellular contributors to vascular regulation reorganize under PV suppression. We predicted arterial diameter changes from PV+ and PV− cell activity using a HRF fitted with a gamma function at the baseline and post-DCZ injection in awake PV-hM4Di(Gi) mice (Supplemental Figure 4). For each neuron, we computed the Pearson correlation between measured and predicted arterial diameter (Figure 7(e)). Neurons were selected from 20 recordings in 9 animals if the correlation exceeded 0.2 at either baseline or post-DCZ.

For PV+ cells, two clusters were identified based on baseline correlations: the first cluster (*n* = 13 cells) had a higher average correlation (0.24 ± 0.03), while the second cluster (*n* = 12 cells) had a lower average correlation (0.04 ± 0.06). Following DCZ injection, the first cluster’s correlation decreased to 0.08 ± 0.10 (*p* < 0.01), whereas the second cluster increased to 0.36 ± 0.12 (*p* < 0.01). A similar pattern was observed for PV− cells: baseline correlations revealed a high-correlation cluster (*n* = 51 cells, 0.27 ± 0.05) and a low-correlation cluster (*n* = 34 cells, 0.04 ± 0.07). After DCZ, correlations in the high-correlation cluster declined to 0.09 ± 0.12 (*p* < 0.001), whereas those in the low-correlation cluster increased to 0.32 ± 0.11 (*p* < 0.001). Control analyses, in which 20 cells were randomly selected from each population, showed no clustering (Supplemental Figure 5), confirming that the observed patterns were not due to random noise. Comparison of the average HRF kinetics within these clusters revealed shifts in parameters such as time-to-peak and peak amplitude following PV suppression, suggesting a reorganization of the neurovascular coupling response (Supplemental Figure 6). Convolving the EEG BLPs with the condition-specific population HRFs derived from all recorded neurons did not recover the reduced correlation between gamma-band oscillations and CBF in the post-suppression state (Supplemental Figure 7). These findings suggest a state-dependent reorganization of neurovascular control: as network excitability increases, one group of neurons (Cluster 1) loses its vascular influence while another group (Cluster 2) takes over this role.

## Discussion

Our findings reveal that PV interneurons shape resting-state neurovascular dynamics by differentially regulating network excitability, frequency-specific neural activity, and cerebrovascular responses. Using chemogenetic suppression of PV cells, we examined their contribution to neuronal activity, EEG power spectra, CBF, and vascular dynamics during ongoing activity. We confirmed the effective modulation of PV cells via DCZ activation of inhibitory DREADDs led to network hyperexcitability (Figure 2). PV suppression reduced high-gamma EEG power while increasing low-frequency EEG power (Figure 3), accompanied by a robust increase in basal CBF and CBF fluctuations (Figure 4). Single-vessel 2PLSM imaging further supported these effects, showing enlarged basal arterial diameters and increased diameter fluctuations in middle cortical layers (Figure 5). Most importantly, PV suppression disrupted the tight coupling between gamma-band activity and spontaneous slow CBF fluctuations (Figure 6). At the cellular level, PV suppression significantly increased apparent neuronal synchrony without altering its coupling to spontaneous arterial responses (Figure 7(a)–(d)). Remarkably, single-cell analysis revealed a redistribution of neurovascular control across distinct neuronal subpopulations, with one group losing vascular influence as another compensatory group emerged, maintaining vascular regulation through mechanisms independent of gamma coordination (Figure 7(e)).

PV suppression significantly decreased the correlation between gamma-band EEG power and CBF, suggesting that PV cell activity is essential for maintaining the canonical gamma-CBF relationship during resting state. This disruption occurred in the context of pronounced network hyperexcitability: excitatory cells showed elevated activity, and high-gamma power was reduced. Similar phenomena have been reported in Alzheimer’s disease (AD) conditions, where amyloid-β pathology disrupts excitation-inhibition balance, resulting in hyperactivity, hypersynchrony, and impaired oscillations.^33–35^ Such functional disruptions often precede structural pathology, underscoring their role as a potential early biomarker of circuit dysfunction.^36^ We were surprised not to observe a larger reduction in gamma power following PV suppression. This outcome may reflect compensatory increases in excitatory cell activity, as increased spiking activity could elevate gamma power through the additional energy generated.

The hemodynamic changes following PV suppression likely result from elevated excitatory activity, as PV interneurons themselves are not major sources of vasoactive molecules. In contrast, excitatory neurons release vasoactive agents such as prostaglandin E_2_ that promote vasodilation and can also directly dilate blood vessels through mechanisms independent of prostaglandin E2.^37,38^ This hyperexcitability likely amplifies excitatory-neuron mediated vasoactive signaling, sustaining the observed increases in basal CBF and arterial diameter. Together, these findings highlight the critical role of PV interneurons in maintaining both baseline cerebral perfusion and normal patterns of NVC, thereby preserving the high dynamic range necessary to support task-specific neural computation while protecting the brain from the metabolic and mechanical stress of chronic vasodilation. While this acute PV suppression results in an increase in basal CBF, we reason this does not represent a protective hyperperfusion, but rather a state of hemodynamic instability.

While clinical AD is defined by hypoperfusion, multiple studies show that regional hyperperfusion, particularly in cognitive-associated regions, often precede global decline during the preclinical stages.^39^ This early increase in CBF likely reflects a compensatory or pathological elevation of neural activity, potentially driven by PV interneuron dysfunction, to maintain brain oxygenation in response to incipient capillary dysfunction or increased metabolic demand.^40,41^ Specifically, evidence suggests that at-risk individuals and those with early mild cognitive impairment show increased CBF in frontal and hippocampal regions, which correlates with memory preservation before the transition to the hypoperfusion characteristic of late-stage disease.^39^ Data-driven multifactorial analyses of late-onset AD cohorts similarly highlight that cerebrovascular dysregulation is the earliest detectable pathological change, preceding major amyloid accumulation and global hypoperfusion.^42^ Therefore, our model of PV suppression-induced hyperperfusion aligns with the “compensatory hyperperfusion” phase observed in early AD pathology prior to neurovascular exhaustion.

Given the established role of gamma oscillations in local computation and feedforward signaling, the observed shift toward slower oscillations with elevated delta-alpha power likely reflects the emergence of synchronous population events under reduced inhibition.^16–18^ Consistent with this, PV suppression increased apparent neuronal synchrony, as measured from Ca^2+^-inferred spiking activity. Disinhibition may promote the simultaneous recruitment of larger neuronal ensembles, increasing pairwise correlations across the network. While our synchrony metric, derived from deconvolved Ca^2+^ fluorescence signals, lacks the temporal precision of extracellular recordings, and frequency-specific EEG oscillations were computed from leads placed in the opposite hemisphere, it still provides reliable relative changes across conditions. The observed hypersynchrony aligns with previous findings in AD mouse model and epileptiform activity, where PV dysfunction or excessive excitation leads to paradoxical large-scale synchrony.^28–30^

Interestingly, the coupling between apparent neuronal synchrony and spontaneous arterial diameter fluctuations did not increase despite pronounced network hyperexcitability following PV suppression, establishing tighter constraints on the nature of the synchrony that drives strong vascular responses. At the same time, the disruption of canonical gamma-CBF coupling indicates a breakdown of established frequency-specific neurovascular relationships rather than a shift to alternative oscillatory drivers. HRF-based single-cell analysis suggested a state-dependent reorganization wherein neurovascular control redistributed from one cellular ensemble (Cluster 1) to another (Cluster 2) across both PV+ and PV− populations. Under baseline conditions, Cluster 1 neurons dominated vascular regulation within a gamma-coordinated network framework, as evidenced by strong gamma-CBF coupling. Following PV suppression, these cells lost their vascular influence while Cluster 2 neurons, previously weakly coupled to vessel, emerged as the primary vascular regulators. Critically, this compensatory takeover maintained single-neuron vascular prediction but likely operated independently of gamma-band coordination, which could explain the paradox of preserved cellular-level coupling alongside disrupted population-level gamma-CBF relationships. This reorganization likely reflects engagement of alternative coupling mechanisms, potentially operating through lower-frequency activity bands. Furthermore, the loss of population-level coordination may signal a shift into a non-linear regime; while PV interneurons typically “linearize” neurovascular output by gating pyramidal cell bursts, their suppression likely induces hemodynamic saturation or neural disinhibition, where vascular responses no longer scale proportionally with high-frequency neural inputs.

In summary, our findings suggest that PV interneurons play an important role in regulating the specificity and stability of resting-state neurovascular coupling across brain states. By showing that inhibitory control constrains which neural rhythms and cellular ensembles effectively drive CBF, this work highlights how alterations in excitation-inhibition balance can fundamentally reshape hemodynamic signals. Our findings provide a cellular framework for understanding one of the primary components underlying the fMRI BOLD signal – vascular dynamics – and offer important considerations for the interpretation of vascular-based imaging in conditions marked by PV dysfunction, including epilepsy, AD, and schizophrenia. Future studies should determine the molecular identity and firing dynamics of Cluster 1 versus Cluster 2 neurons to elucidate whether possible compensatory vascular control operates through low-frequency oscillations or cell-type-specific vasoactive signaling pathways independent of gamma coordination.

## Supplemental Material

sj-pdf-1-jcb-10.1177_0271678X261462775 – Supplemental material for Parvalbumin interneurons contribute to spontaneous hemodynamic fluctuations Supplemental material, sj-pdf-1-jcb-10.1177_0271678X261462775 for Parvalbumin interneurons contribute to spontaneous hemodynamic fluctuations by Adiya Rakymzhan, Mitsuhiro Fukuda and Alberto L. Vazquez in Journal of Cerebral Blood Flow & Metabolism
