# Supplementary material for: Parvalbumin interneurons contribute to spontaneous hemodynamic fluctuations
Source: J Cereb Blood Flow Metab. 2026 Jul 15:0271678X261462775. Online ahead of print. doi: 10.1177/0271678X261462775 (PMC13447528; doi:10.1177/0271678X261462775)
Supplement: Supplementary file 1 [file sj-pdf-1-jcb-10.1177_0271678X261462775.pdf]

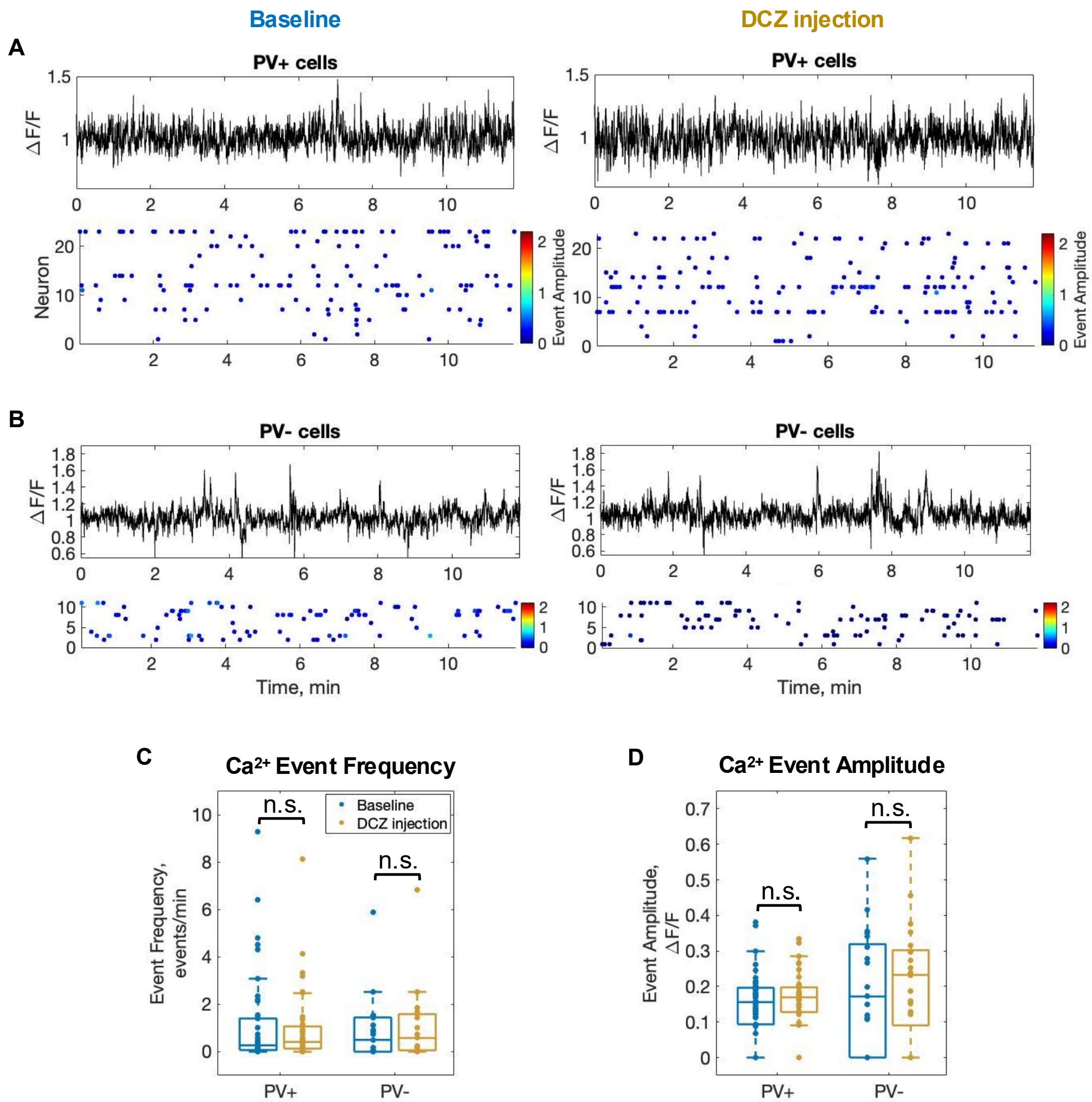

Supplemental Figure 1. DCZ injection does not affect neuronal activity in naïve mice. A. Time profiles of Ca^{2+} fluorescence changes (black traces) in an individual PV+ cell in a PV-mCherry mouse (from a control group) during ongoing activity at baseline and post-DCZ injection. Below are the raster plots of Ca^{2+} activity in PV+ cell population from a single animal at baseline and post-DCZ injection. Each dot represents a Ca^{2+} event and each raw represents an individual neuron. The dot color encodes an event amplitude. **B**. Similar analysis as in A but for PV- cells. **C**. Summary of Ca^{2+} event frequency and **D**. Ca^{2+} event amplitude in 92 PV+ cells and 42 PV- cells identified from 3 recordings in 3 animals (control group). No significant differences between baseline and post-injection are detected for event frequency or event amplitude in any neuronal sub-population. Non-significant differences are denoted by n.s.

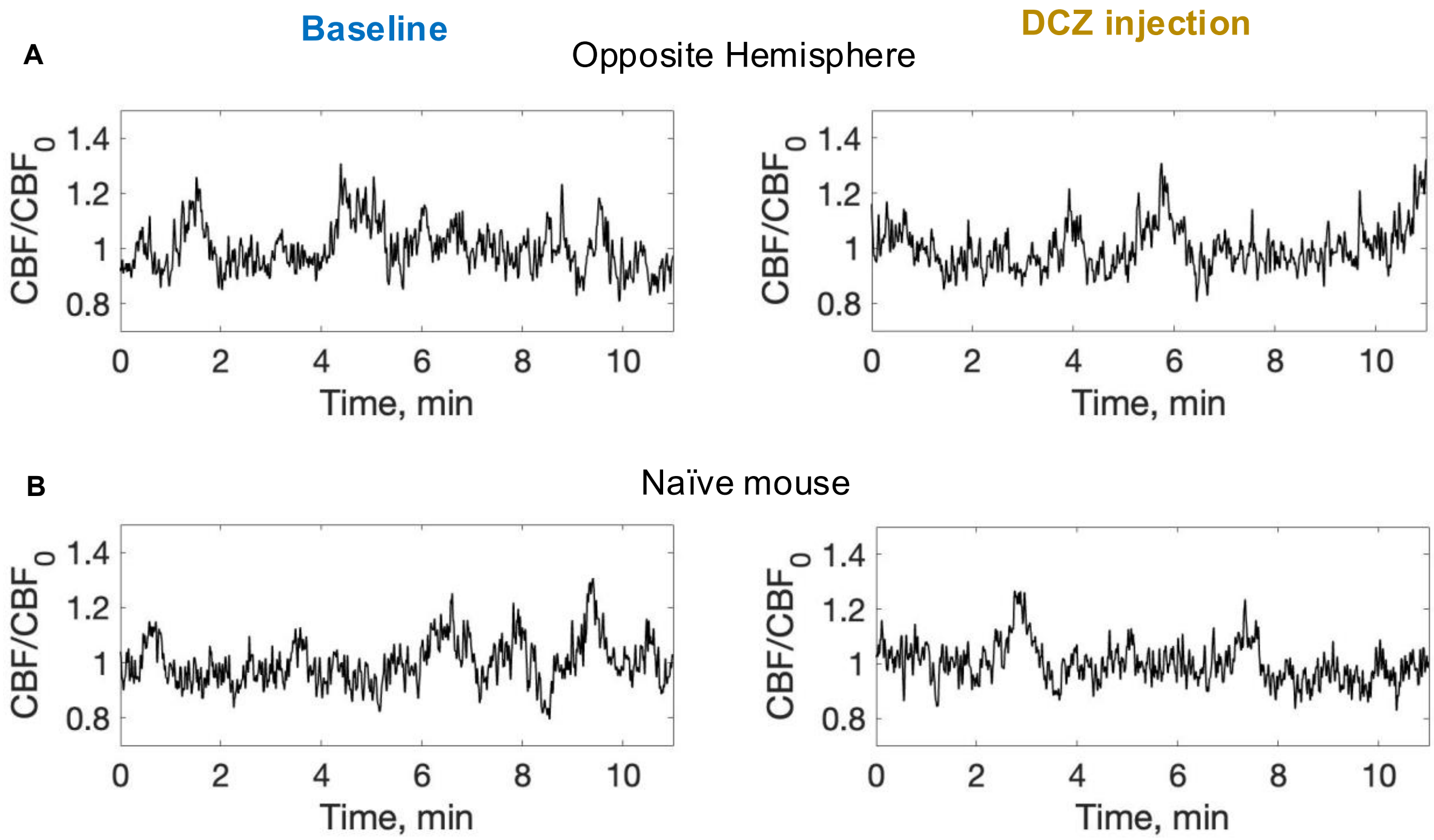

Supplemental Figure 2. DCZ injection does not change CBF in the control group. A. Example of spontaneous CBF time profiles in the opposite hemisphere of a PV-hM4Di(Gi) mouse and **B**. in a PV-mCherry (naïve) mouse at the baseline and post-DCZ injection.

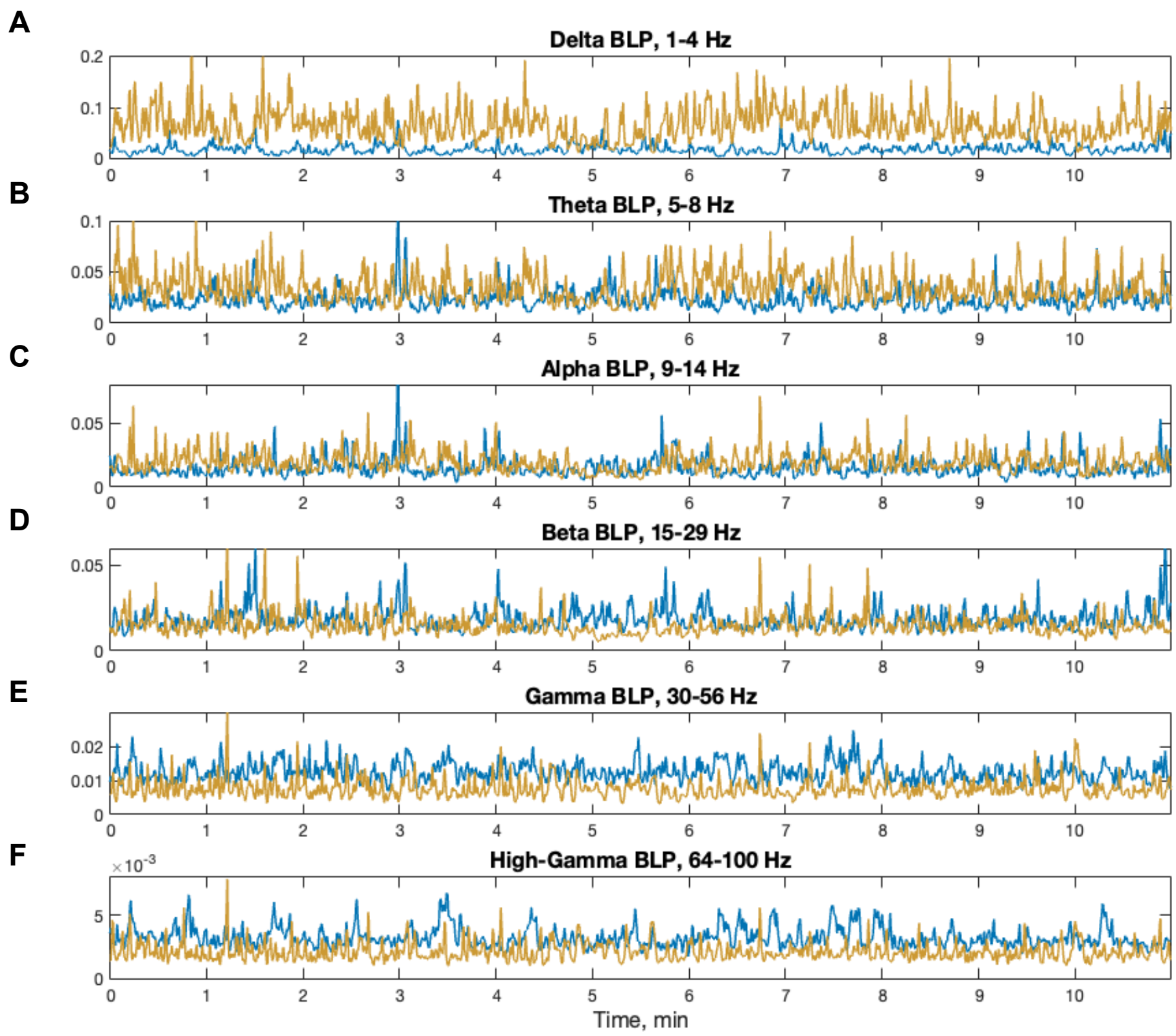

Supplemental Figure 3. Effect of suppressing PV neuron activity on time-resolved band-limited EEG power. Example time courses of time-resolved BLPs at baseline (blue) and post-DCZ injection (yellow) in a PV-hM4Di(Gi) mouse.

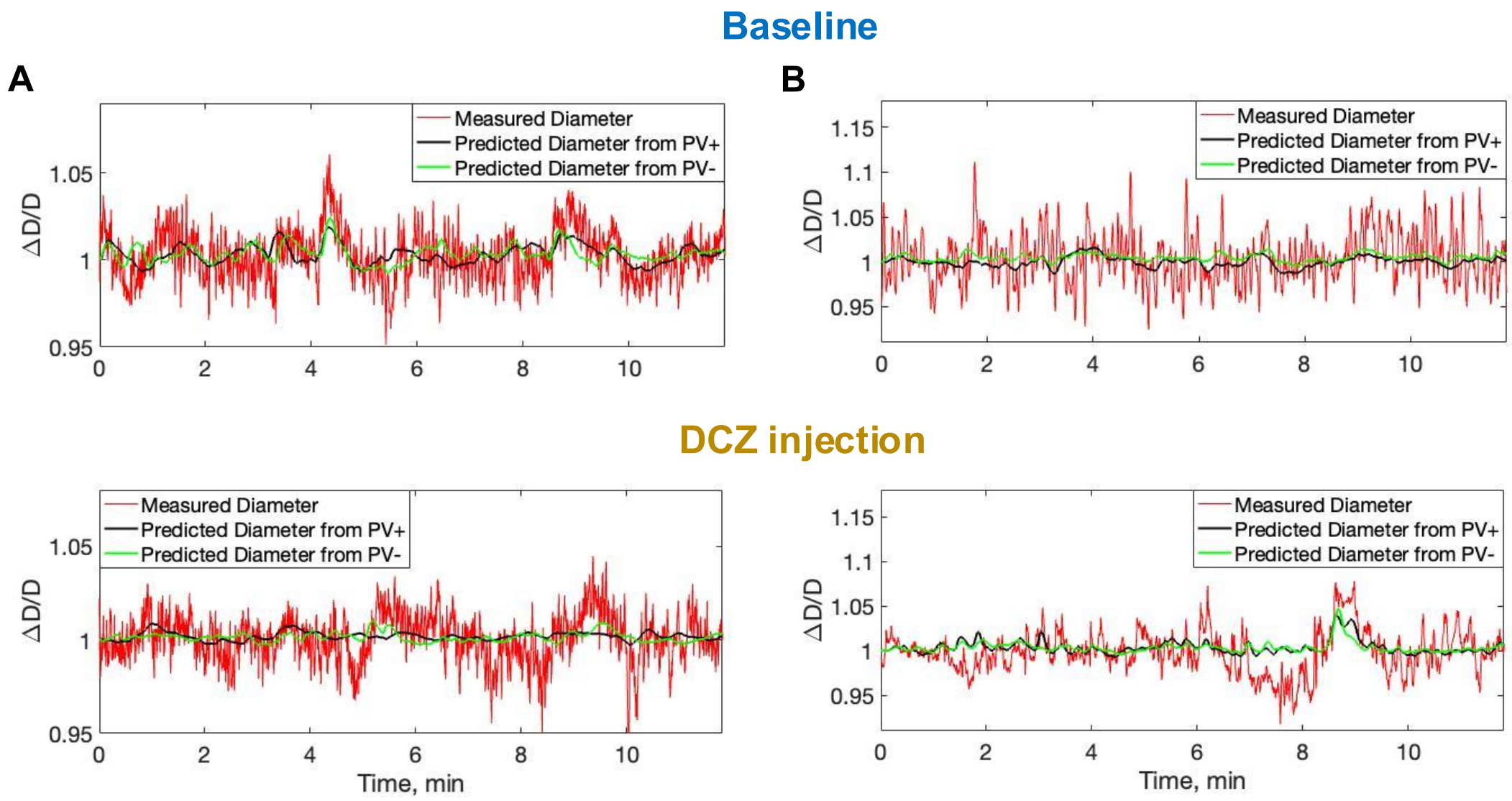

Supplemental Figure 4. Measured vs. predicted arterial diameter changes. A. Example time profiles showing measured arterial diameter changes (red), predicted diameter changes based on activity from a single PV+ cell (black), and predicted diameter changes based on activity from a single PV- cell (green) for a single vessel in a PV-hM4Di(Gi) mouse at baseline and post-DCZ injection. In this example, the correlation between measured and predicted (from both PV+ and PV-) diameters decreased post-DCZ injection. **B**. Similar example as in B, where the correlation between measured and predicted (from both PV+ and PV-) diameters increased post-DCZ injection.

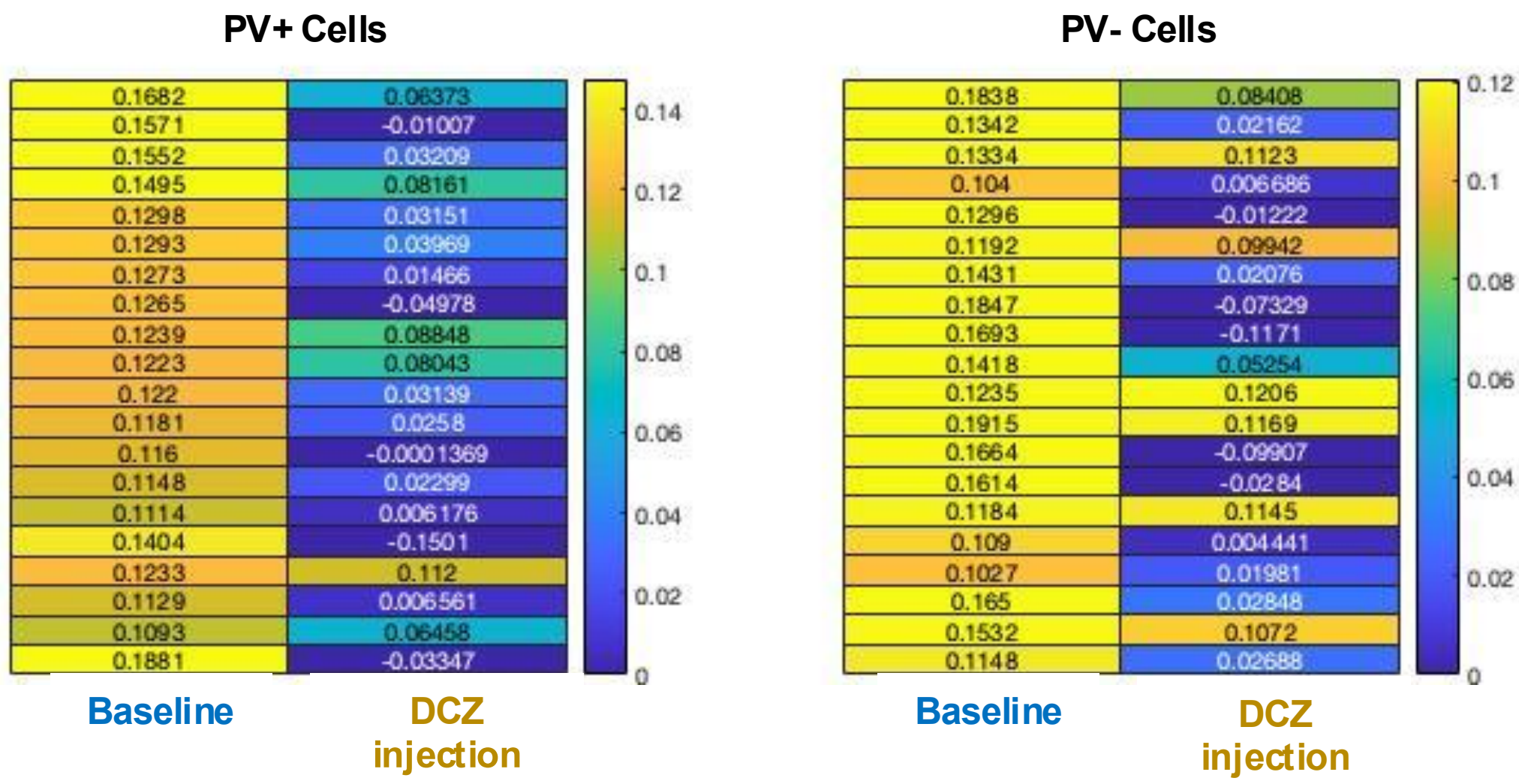

Supplemental Figure 5. The observed cell clustering was not the effect of noise. Heatmaps depicting Pearson correlation coefficients between arterial diameter changes and predictions based on activity of randomly selected 20 PV+ or PV− cells, both at baseline and post-DCZ injection. Each row represents a single neuron, with rectangle colors indicating the magnitude of the correlation. No definitive cluster were identified for either neuronal subpopulation (PV+ and PV−).

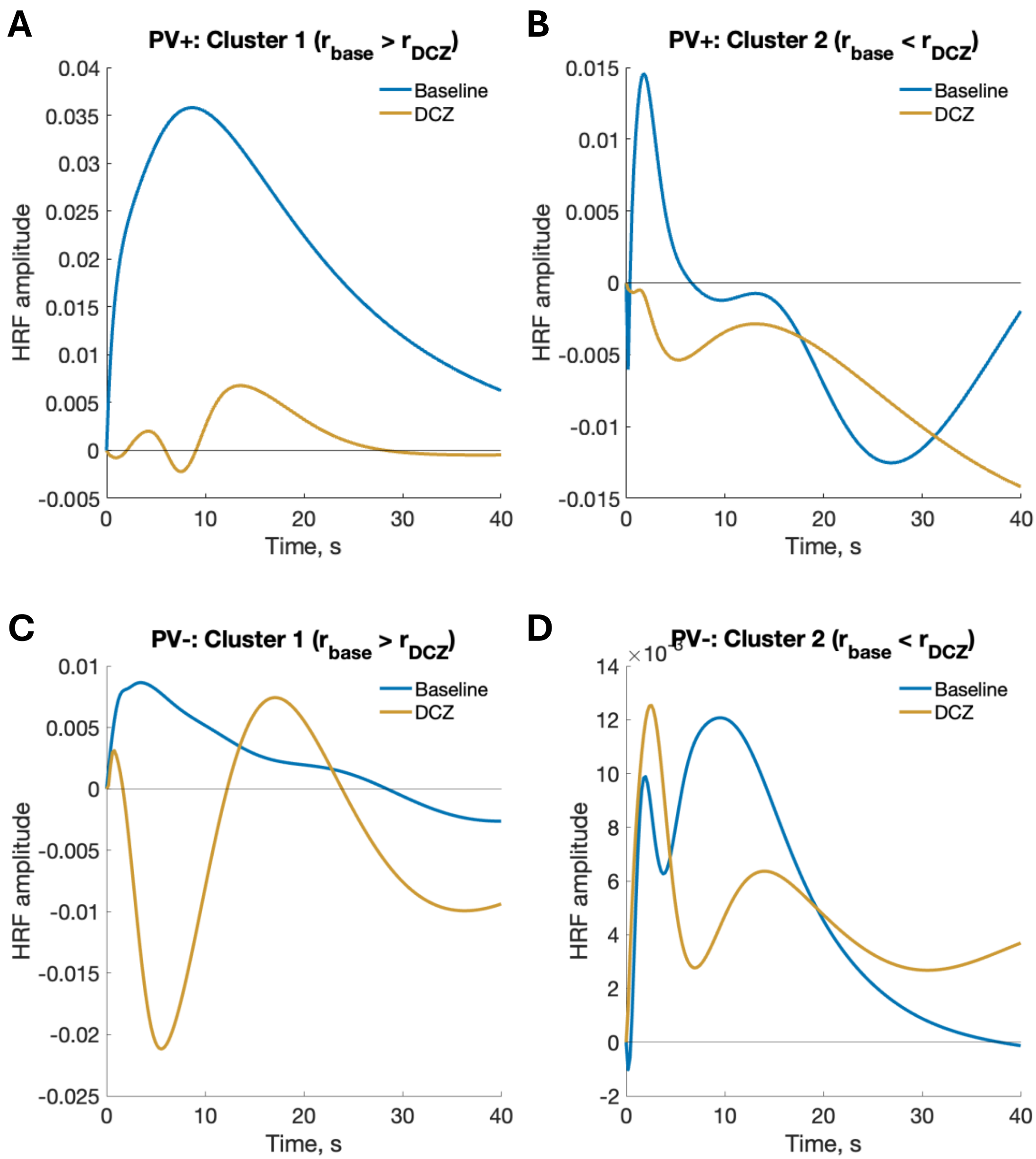

Supplemental Figure 6. Comparison of the average HRF kinetics within cell clusters revealed morphological transformation following PV suppression, suggesting a reorganization of the neurovascular coupling response. Average HRF of **A.** PV+ cells from Cluster 1, **B.** PV+ cells from Cluster 2, **C.** PV- cells from Cluster 1, **D.** PV- cells from Cluster 2 at baseline and post-DCZ injection.

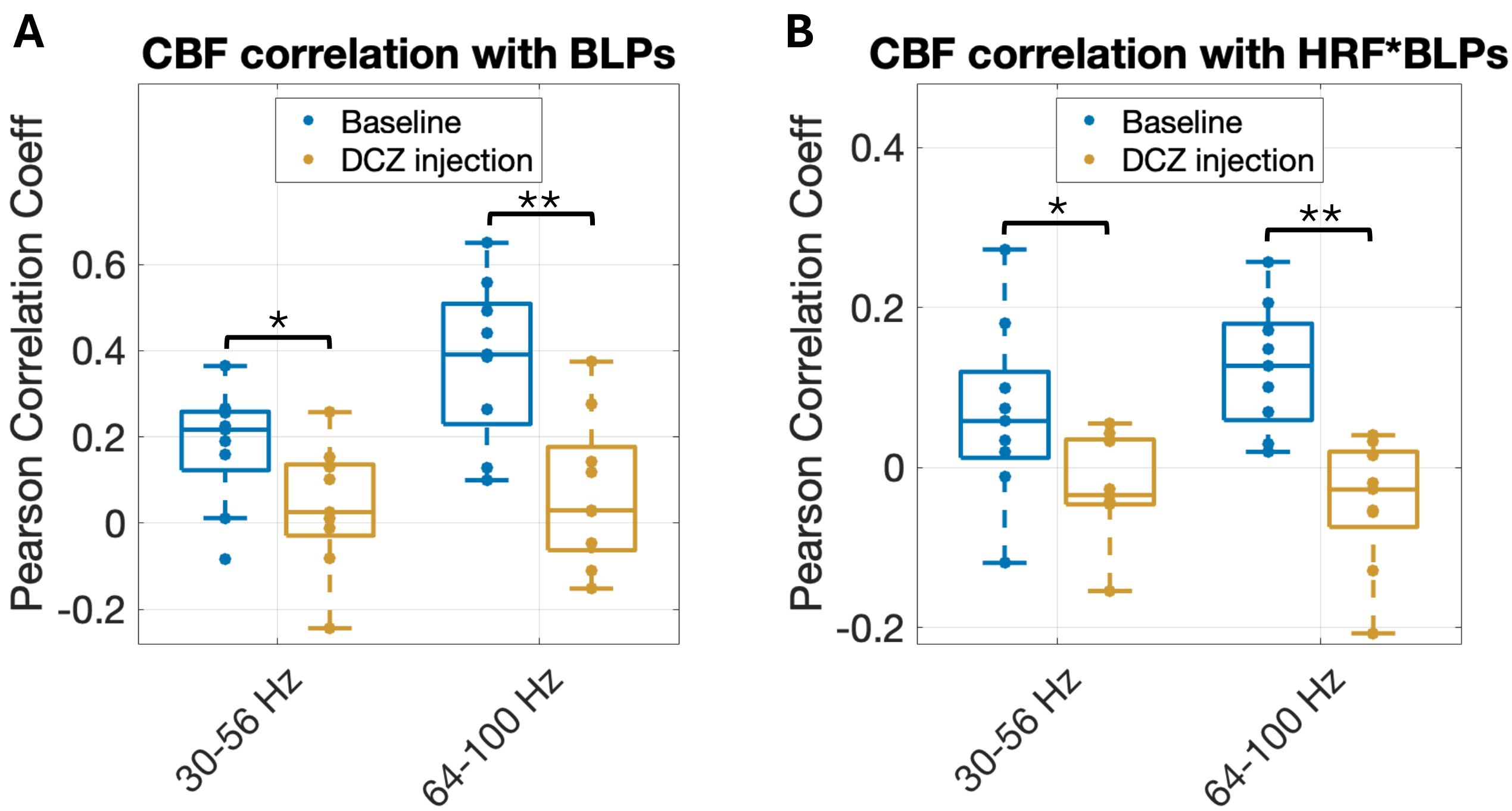

Supplemental Figure 7. Comparison of the average correlations between CBF and EEG BLPs vs. those between CBF and the global HRF convolved with EEG BLPs. A. Zoom-in of the average CBF correlation with gamma and high-gamma BLP from Figure 6C; **B.** Average CBF correlation with gamma and high-gamma BLP convolved with the 'global' population HRF derived from all 2PLSM-recorded neurons at baseline and post-DCZ injection.
